# The Influence of Intracellular Glutathione Levels on the Induction of Nrf2-Mediated Gene Expression by α-Dicarbonyl Precursors of Advanced Glycation End Products

**DOI:** 10.3390/nu14071364

**Published:** 2022-03-24

**Authors:** Liang Zheng, Katja C. W. van Dongen, Wouter Bakker, Ignacio Miro Estruch, Ivonne M. C. M. Rietjens

**Affiliations:** Division of Toxicology, Wageningen University and Research, Stippeneng 4, 6708 WE Wageningen, The Netherlands; katja.vandongen@wur.nl (K.C.W.v.D.); wouter.bakker@wur.nl (W.B.); ignacio.miroestruch@wur.nl (I.M.E.); ivonne.rietjens@wur.nl (I.M.C.M.R.)

**Keywords:** α-dicarbonyl compounds, glutathione, methylglyoxal, glyoxal, 3-deoxyglucosone, Nrf2

## Abstract

α-Dicarbonyl compounds, particularly methylglyoxal (MGO), glyoxal (GO), and 3-deoxyglucosone (3-DG), are highly reactive precursors for the formation of advanced glycation end products (AGEs). They are formed in vivo and during food processing. This study aimed to investigate the role of intracellular glutathione (GSH) levels in the induction of Nrf2-mediated gene expression by α-dicarbonyl compounds. The reactions between α-dicarbonyl compounds (MGO, GO, and 3-DG) and GSH were studied by LC-MS in a cell-free system. It was shown that these three α-dicarbonyl compounds react instantaneously with GSH, with the GSH-mediated scavenging decreasing in the order MGO > GO > 3DG. Furthermore, in a cell-based reporter gene assay MGO, GO, and 3-DG were able to induce Nrf2-mediated gene expression in a dose-dependent manner. Modulation of intracellular GSH levels showed that the cytotoxicity and induction of the Nrf2-mediated pathway by MGO, GO and 3-DG was significantly enhanced by depletion of GSH, while a decrease in Nrf2-activation by MGO and GO but not 3-DG was observed upon an increase of the cellular GSH levels. Our results reveal subtle differences in the role of GSH in protection against the three typical α-dicarbonyl compounds and in their induction of Nrf2-mediated gene expression, and point at a dual biological effect of the α-dicarbonyl compounds, being reactive toxic electrophiles and -as a consequence- able to induce Nrf2-mediated protective gene expression, with MGO being most reactive.

## 1. Introduction

α-Dicarbonyl compounds are by-products from cellular metabolism formed in vivo by glycation, degradation of glycolytic intermediates, and lipid peroxidation [1,2]. They are also found in various foods and beverages, in which they are mainly formed by caramelization and Maillard reactions during thermal processing and storage of food [3,4]. Among the α-dicarbonyl compounds, also so-called α-oxoaldehydes, methylglyoxal (MGO), glyoxal (GO), and 3-deoxyglucosone (3-DG) are the major precursors for advanced glycation end products (AGEs), the formation of which has been associated with many chronic diseases [5,6]. Typical concentrations of MGO, GO, and 3-DG are 1–4 μM in cells, but these concentrations are elevated under pathological circumstances such as metabolic disorders [7,8]. The production of α-dicarbonyl compounds during thermal processing and storage of food further increases human exposure to these compounds or the resulting AGEs via dietary intake. Increased in vivo concentrations of these α-dicarbonyl compounds may result in a dysfunctional metabolic state also called dicarbonyl stress, which causes an increase in the production of reactive oxygen species (ROS) and proinflammatory cytokines and induces loss of mitochondrial membrane potential in different cell types [9,10,11]. Especially, under dicarbonyl stress, the pronounced electrophilic reactivity of the α-dicarbonyl compounds facilitates their spontaneous reactions with nucleophilic reaction sites of proteins to form AGEs causing protein damage and aggregation, which contributes to cell and tissue dysfunction and eventually may play a role in the development of diabetes mellitus, cardiovascular diseases, and a variety of age-related diseases, such as Parkinson’s disease and Alzheimer’s disease [12,13,14]. Understanding the underlying mechanisms by which cells are affected by or can be protected against α-dicarbonyl compounds is therefore of particular importance.

**Figure 1:**
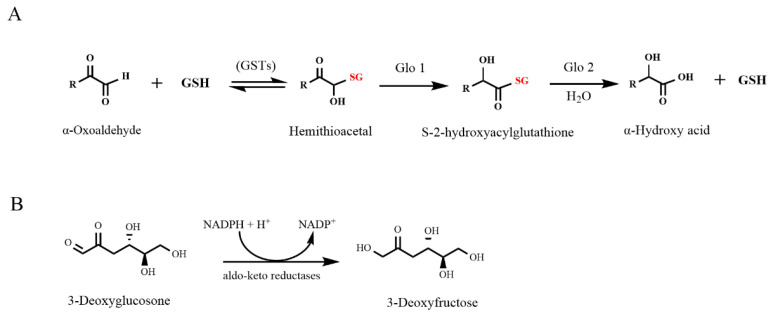
Major intracellular detoxification pathways for α-dicarbonyl compounds including (**A**) detoxification of the α-dicarbonyl compounds (mainly MGO and GO) by the glyoxalase system and (**B**) detoxification (mainly for 3-DG) by NADPH-dependent aldo-keto reductases. GSH: glutathione, GSTs: glutathione-S-transferases, MGO: methylglyoxal, GO: glyoxal, 3-DG: 3-deoxyglucosone, Glo1: glyoxalase 1, Glo2: glyoxalase 2.

Several metabolic pathways in the human cells are known to antagonize the formation of AGEs mediated by α-dicarbonyl compounds. The glyoxalase system, which is highly dependent on glutathione (GSH), has been reported to provide a major detoxification pathway for MGO and GO in cells [15]. MGO and GO can react with the nucleophilic thiol group of GSH both non-enzymatically and in a reaction catalyzed by glutathione-S-transferases (GSTs) to form a hemithioacetal which can be further converted by glyoxalase 1 and 2 (Glo1 and Glo2) to the corresponding α-hydroxy acid (Figure 1A) [16,17,18]. 3-DG is mainly detoxified to 3-deoxyfructose by NADPH-dependent aldo-keto reductases (Figure 1B) [7]. Previous studies have confirmed that GSH plays a crucial role in the detoxification of MGO and GO [18,19], while for 3-DG this role remains uncertain. The aim of the present study was to compare the role of intracellular GSH in counteracting the cellular effects of the three reactive α-dicarbonyls, including cytotoxicity and the induction of the nuclear factor erythroid 2 p45-related factor 2 (Nrf2)-mediated gene expression.

Nrf2 is an essential transcription factor regulating the expression of genes containing an electrophile-responsive element (EpRE) responsible for the induction of a wide range of protective enzymes involved in the synthesis of GSH and protection of cells against electrophilic as well as oxidative stress [15,20]. Due to the reactive electrophilic activity of the α-dicarbonyl compounds, possible reversible binding of the dicarbonyls to reactive cysteine residues in the regulatory inhibitory protein Keap1 (Kelch-like erythroid-cell-derived protein with CNC homology-associating protein 1), the major regulator of Nrf2, may result in the release of Nrf2 from Keap1, leading to nuclear translocation of Nrf2 and the activation of EpRE-mediated gene transcription [7]. Some studies have reported that pretreatment of cells with Nrf2 activators, such as carnosic acid and naringenin, promoted the levels of intracellular GSH by increasing the expression of cystine/glutamate transporter and glutamyl-cysteine ligase (GSH synthesis rate-limiting enzyme) via the Nrf2 signaling pathway, contributing to the attenuation of MGO-induced cytotoxicity in SH-SY5Y cells [21,22]. However, the direct involvement of Nrf2 in the adaptive cellular response to exposure to α-dicarbonyl compounds has not been fully elucidated, and the potential differences in the induction of the Nrf2-mediated transcriptional pathway by the three α-dicarbonyl compounds and the influence on this process by cellular GSH levels still needs to be clarified. In this study, the scavenging efficacy of GSH on the three α-dicarbonyl compounds was evaluated in an in vitro cell-free system. Furthermore, the influence of intracellular GSH levels on cytotoxicity and on the Nrf2-mediated gene expression induced by the selected α-dicarbonyl compounds was studied using the U2OS Nrf2 reporter gene cells [23], either without or upon modulation of their intracellular GSH levels.

## 2. Materials and Methods

### 2.1. Chemicals and Reagents

MGO (40% in water), GO (40% in water), L-glutathione reduced (GSH, purity ≥ 98%), L-glutathione oxidized (GSSG, purity ≥ 98%), N-acetyl-L-cysteine (NAC, purity ≥ 99%), L-buthionine-sulfoximine (BSO, purity ≥ 97%), 2′,7′-dichlorofluorescin diacetate (DCFDA, purity ≥ 97%), and *tert*-butyl hydroperoxide (TBHP, 70% in water) were purchased from Sigma-Aldrich (St. Louis, MO, USA). 3-Deoxyglucosone (3-DG, purity > 99%) was obtained from Toronto Research Chemicals (Toronto, ON, Canada). Trichloroacetic acid (TCA, purity ≥ 98%) and acetic acid (purity ≥ 99%) were purchased from Merck (Darmstadt, Germany). Acetonitrile (LC-MS grade) was purchased from Biosolve BV (Valkenswaard, The Netherlands). Formic acid (purity ≥ 99%) was purchased from VWR CHEMICA (Amsterdam, The Netherlands). WST-1 reagent was obtained from Roche (Mannheim, Germany). Dulbecco’s Modified Eagle Medium/Nutrient Mixture F-12 (DMEM/F-12) with GlutaMAX supplement cell culture medium (with and without phenol red), penicillin/streptomycin, Hanks’ balanced salt solution (HBSS) and phosphate buffered saline (PBS) were purchased from Gibco (Paisley, UK). Foetal calf serum (FCS) was obtained from Bodinco (Alkmaar, The Netherlands). Trypsin, nonessential amino acids (NEAA), and geneticin (G418) were obtained from Invitrogen Corporation (Breda, The Netherlands). Ultrapure water was prepared by a Milli-Q system (Millipore, MA, USA). All other reagents in this study were of analytical grade or purer.

### 2.2. Cell Lines

The Nrf2 CALUX cells (BioDetection Systems, Amsterdam, The Netherlands) are human osteosarcoma U2OS cells, which were stably transfected with a reporter construct carrying a luciferase reporter gene under transcriptional control of four EpREs [23].

The Cytotox CALUX cells (BioDetection Systems, Amsterdam, The Netherlands) are human osteosarcoma U2OS cells stably transfected with a reporter construct carrying a luciferase reporter gene under transcriptional control of a constitutive promoter [24]. The reporter construct was generated by inserting the luciferase gene into the multiple cloning site of the pSG5-neo vector [25]. These cells have an invariant luciferase expression and a decrease in luciferase activity therefore indicates a cytotoxic effect. Besides, an increased luciferase activity in these cells may indicate stabilization of luciferase reporter protein without underlying increased expression of the gene [26].

Both cell lines were cultured in DMEM/F12 GlutaMAX medium containing 7.5% FCS, 1% NEAA and 0.3% penicillin/streptomycin in a humidified incubator of 5% CO_2_ at 37 °C. 200 μg/mL G418 was added to the culture medium once a week to maintain the selection pressure [23].

### 2.3. Kinetic Study of the Reaction between α-Dicarbonyl Compounds and GSH

Stock solutions of MGO, GO, 3-DG, GSH, and GSSG were all prepared in 25 mM potassium phosphate (pH = 7.4). To a starting solution of GSH (final concentration 0.5 mM) were added different volumes of the stock solution of MGO, GO, or 3-DG to give final concentration of 0.5 mM or 5 mM, followed by incubation of the resulting solution at 37 °C in a water bath for 0 h, 0.5 h, 1 h, 2 h, 4 h and 6 h. At each time point, 4 μL acetic acid was added to the collected samples (196 μL) to stabilize GSH and its related adducts. Samples were immediately stored at −80 °C until analysis by LC-MS as further described in 2.4 and 2.5.

### 2.4. LC-TOF-MS Analysis

An Agilent 1200 LC system coupled with a Bruker micro-TOF mass spectrometer was used to qualitatively detect the reaction products between α-dicarbonyl compounds and GSH. A Phenomenex Luna Omega Polar C18 (100 mm × 2.1 mm, 1.6 μm) column was employed during the experiment with a flow rate of 0.18 mL/min. The mobile phase was composed of A (ultrapure water with 0.1% formic acid) and B (acetonitrile with 0.1% formic acid) with the following gradient elution: 0–2 min, 100%A; 2–7 min 100–40%A; 7–8.5 min 40–20%A; 8.5–8.7 min 20–100%A; 8.7–24 min 100%A. The injection volume was 1 μL. Mass spectrometric analysis was performed in the positive electrospray ionization mode with mass spectra acquired from *m/z* 100 to 1500. The mass parameters were: capillary voltage, −4500 V; nebulizing gas pressure, 1.2 bar; drying gas flow, 8 L/min and drying temperature, 200 °C.

### 2.5. LC-TQ-MS Analysis

A Shimadzu Nexera XR LC-20AD XR UHPLC system coupled with a Shimadzu 8040 triple quadrupole mass spectrometer with electrospray ionization (ESI) interface was applied for quantification of GSH in the samples. Chromatographic separation was achieved using the same column as used in the LC-TOF-MS analysis. Ultrapure water containing 0.1% formic acid (A) and acetonitrile with 0.1% formic acid (B) were used as mobile phase at a flow rate of 0.2 mL/min. The following gradient was used: 0–1 min, 100%A; 1–5 min 100–35%A; 5–7.5 min 35%A; 7.5–7.6 min 35–100%A; 7.6–18 min 100%A. The instrument was operated in positive ionization mode with multiple reaction monitoring (MRM) for quantification. The mass parameters were: nebulizing gas flow, 3.0 L/min; drying gas flow and heating gas flow, 10.0 L/min; interface temperature, 300 °C; and heat block temperature, 400 °C. GSH was monitored at the [M + H]^+^ of precursor to product ion transitions of *m*/*z* 307.90 → 179.05 (collision energy (CE)  =  −12 eV), 307.90 → 76.10 (CE  =  −25 eV), 307.90 → 162.05 (CE  =  −16 eV), and 307.90 → 84.05 (CE  =  −21 eV). GSSG was monitored at the [M + H]^+^ of precursor to products of *m*/*z* 613.15 → 355.05 (CE  =  −22 eV), 613.15 → 231.00 (CE  =  −22 eV), 613.15 → 484.15 (CE  =  −22 eV) and 613.15 → 177.10 (CE  =  −30 eV). Quantification of GSH in the samples was achieved via calibration curves made using the reference compound. The remaining GSH (%) was calculated using the following equation: Remaining GSH (%) = detected amount of GSH in test samples/original amount of GSH in the samples × 100.

### 2.6. Nrf2 CALUX Assay

The induction of Nrf2-mediated gene expression by α-dicarbonyl compounds was tested by measuring the luciferase activity in the Nrf2 CALUX cells. Briefly, Nrf2 CALUX cells (2 × 10^4^ cells/well) were seeded into white opaque 96-well plates (Greiner Bio-one) and incubated for 24 h. Next, culture medium was refreshed and cells were incubated for another 24 h to allow them to form a confluent monolayer. The culture medium was then replaced by assay medium (DMEM/F12 without phenol red and supplemented with 5% dextran-coated charcoal-stripped FCS (DCC-FCS) [23]) containing different concentrations of each α-dicarbonyl compound for a continuous 24 h exposure. Eight final concentrations (100, 250, 500, 750, 1000, 1250, 1500 and 1750 μM) of each compound were tested. The three α-dicarbonyl compounds were all dissolved in sterile ultrapure water and added to the cells from 200 times concentrated stock solutions in water. Curcumin at 25 μM was used as the positive control in each plate. After 24 h exposure, cells were carefully washed with ½ PBS and lysed by low salt buffer [27]. Subsequently, the plates were placed on ice for 15 min and then frozen at −80 °C overnight. Afterwards, the plates were thawed and the luciferase activity in relative light units (RLU) was measured using a luminometer (GloMax-Multi Detection System-Promega) after the addition of flash mix [27] to each well. The results were expressed as induction factor (IF) compared to the medium control.

### 2.7. Cytotox CALUX Assay

To investigate whether cytotoxicity or stabilization of the luciferase enzyme (false positive) occurred during the exposure to α-dicarbonyl compounds, a parallel Cytotox CALUX assay was carried out using Cytotox CALUX cells [26]. The assay was performed in the same way as the Nrf2 CALUX assay described above.

### 2.8. Cell Viability Assay

A WST-1 assay was used to evaluate the cytotoxicity of the α-dicarbonyl compounds towards the Nrf2 CALUX cells. In brief, after the same seeding and exposure steps as applied in the Nrf2 CALUX assay, 5 μL of WST-1 solution was added to each well of the 96-well plates. The plates were then further incubated for 1 h after which the absorbance at 440 nm and a reference wavelength at 620 nm was measured using a plate spectrophotometer (Molecular Devices, San Jose, CA, USA, Spectra Max M2). The cell viability was expressed as a percentage of medium control set at 100%.

### 2.9. Measurement of Intracellular ROS Levels

The intracellular ROS levels in Nrf2 CALUX cells were measured using the cell permeant fluorogenic dye 2′,7′-dichlorofluorescin diacetate (DCFDA) [28]. Briefly, Nrf2 CALUX cells (3 × 10^4^ cells/well) were seeded into the wells of black 96-well plates (Greiner Bio-one) and incubated for 24 h. Next, culture medium was removed and cells were loaded with DCFDA by incubation for 45 min at 37 °C with 25 µM DCFDA in HBSS containing 0.4% FCS. The DCFDA containing medium was then replaced by assay medium containing different concentrations of each α-dicarbonyl compound for a continuous 6 h exposure. TBHP at 50 μM was used as the positive control in each plate. After 6 h exposure, the levels of 2′,7′-dichlorofluorescein (DCF), reflecting ROS formation, were measured using a fluorescence microplate reader (Molecular Devices, Spectra Max M2) at an excitation wavelength of 485 nm and an emission wavelength of 535 nm. The results were expressed as fold induction compared to the medium control.

### 2.10. Investigation of the Effects of Altered Intracellular GSH Levels on the Induction of Nrf2-Mediated Gene Expression by the α-Dicarbonyl Compounds

To study the effect of intracellular GSH levels on the induction of Nrf2-mediated gene expression by the α-dicarbonyl compounds, the intracellular GSH level was modulated by the addition of NAC, a precursor of GSH able to increase intracellular levels of GSH [29], and by the addition of BSO to decrease the levels of GSH in cells [30]. Cells were seeded and incubated for 24 h as described above for the Nrf2 CALUX assay. For NAC pre-treatment, after 24 h incubation, the culture medium was replaced by fresh medium and incubated for another 20 h, followed by incubation of the cells with 10 mM NAC for 4 h to allow increase in GSH in the cells. After 4 h of pre-incubation with NAC, culture medium was removed and cells were carefully washed by PBS to remove all extracellular NAC residues before the addition of assay medium containing different concentrations of each α-dicarbonyl compound for a continuous 24 h exposure. Co-exposure of the cells to NAC and α-dicarbonyl compounds was not applied since NAC is known to scavenge α-dicarbonyl compounds [31]. For BSO pre-treatment, after 24 h incubation, the culture medium was replaced by medium containing 100 μM of BSO and incubated for 24 h to decrease the intracellular GSH level. After 24 h of pre-incubation with BSO, culture medium was removed and assay medium containing 100 μM of BSO and different concentrations of each α-dicarbonyl compound were added to cells for 24 h of exposure.

After 24 h of exposure, cells were washed and lysed and the luciferase activity was measured as described above. The WST-1 assay was also carried out in parallel to evaluate the cell viability.

### 2.11. Quantification of the Intracellular GSH Levels

The intracellular levels of GSH were quantified by LC-TQ-MS for control cells and after pre-incubation of cells with 10 mM NAC or 100 μM BSO. Briefly, after the same seeding and pre-incubation steps as described above, the medium in plates was removed and cells were washed with cold PBS, followed by adding 200 μL 2% TCA to stabilize the GSH. The plates were first placed on ice for 15 min and then frozen at −80 °C for more than 6 h before cells were mechanically scraped from the plates. Next, cell lysate from each well was transferred to a centrifuge tube. After being vortexed and centrifuged at 12,000 rpm (13,523× *g*) for 30 min, the supernatant was collected and analyzed by LC-TQ-MS. The intracellular GSH levels were normalized to protein concentrations as quantified by BCA protein assay kits according to the manufacturer’s instructions (Pierce, Thermo Scientific, Waltham, MA, USA).

### 2.12. Statistical Analysis

Data are presented as means ± standard error of the mean (SEM) from at least three independent experiments. An independent-samples *t* test for normally distributed data and a Mann-Whitney U test for non-normally distributed data were applied to compare between any two groups (normality was assessed using the Shapiro-Wilk test) using SPSS 25.0 software (SPSS Inc., Chicago, IL, USA). Statistical significance was defined as *p* < 0.05. Figures were prepared using GraphPad Prism 9 software (San Diego, CA, USA). Chemical structures were drawn by ChemDraw 20.0 (PerkinElmer, Waltham, MA, USA).

## 3. Results

### 3.1. Identification of Reaction Products between α-Dicarbonyl Compounds and GSH by LC-TOF- MS

**Figure 2:**
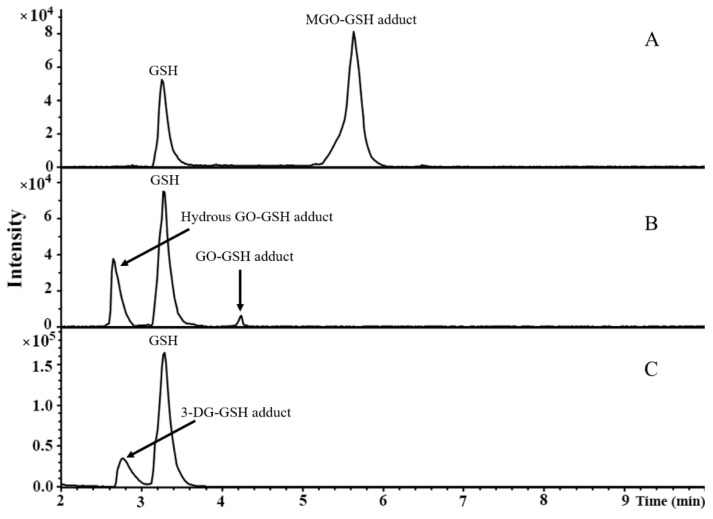
Typical LC-TOF-MS base peak chromatograms of incubations of MGO (**A**), GO (**B**), or 3-DG (**C**) with GSH at a molar ratio of 10:1 for 1 h.

**Figure 3:**
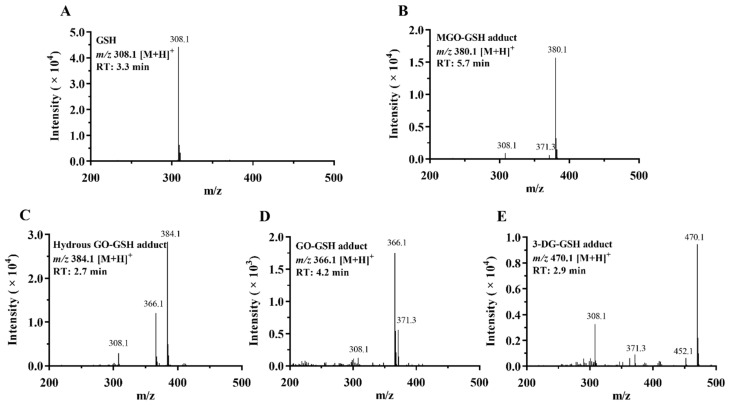
LC-TOF-MS spectra of GSH (**A**), MGO-GSH adduct (**B**), hydrous GO-GSH adduct (**C**), GO-GSH adduct (**D**), and 3-DG-GSH adduct (**E**).

The reaction mixtures of MGO, GO, or 3-DG with GSH were analyzed by LC-TOF-MS. Typical base peak chromatograms are shown in Figure 2. Due to the poor ionization efficiency and stability of dicarbonyls in the ESI source [32], these compounds were not detected. The adducts formed by the reactions between MGO, GO, or 3-DG and GSH at a molar ratio of 10:1 were identified and their mass spectra are shown in Figure 3. After incubation of GSH with MGO for 1 h, a new peak (at 5.7 min) appeared in the chromatogram (Figure 2A) with the molecular ion *m*/*z* 380 [M + H]^+^, which was 72 mass units greater than the molecular ion of GSH *m*/*z* 308 [M + H]^+^, indicating that this peak was GSH conjugated MGO (denoted as MGO-GSH adduct). The fragment ion of this peak *m*/*z* 308 [M − 72 + H]^+^, suggesting the loss of one MGO molecule, further corroborated the formation of the MGO-GSH adduct. Two new reaction products (at 2.7 min and 4.2 min) were observed after the incubation of GSH and GO for 1 h (Figure 2B). The peak at 4.2 min had the molecular ion *m*/*z* 366 [M + H]^+^ and fragment ion *m*/*z* 308 [M – 58 + H]^+^, indicating the loss of one GO molecule, suggesting this product was a GSH adduct of GO (denoted as GO-GSH adduct). The peak at 2.7 min had the molecular ion *m*/*z* 384 [M + H]^+^, which was 18 mass units higher than the *m*/*z* of the GO-GSH adduct (*m*/*z* 366 [M + H]^+^), suggesting a possible hydration of the GO-GSH adduct (denoted as hydrous GO-GSH adduct). By losing a molecule of H_2_O, the fragment ion *m*/*z* 366 [M – 18 + H]^+^ was produced. A previous study showed that the hydrated GO is the dominant form of GO in aqueous solutions [33]. Similarly, a new peak was observed at 2.9 min (Figure 2C) with the molecular ion *m*/*z* 470 [M + H]^+^ and fragment ion *m*/*z* 308 [M – 162 + H]^+^ after incubation of GSH with 3-DG, suggesting it lost one molecule of 3-DG, indicating that this product was a GSH adduct of 3-DG (denoted as 3-DG-GSH adduct).

### 3.2. Kinetic Study of the Reaction between α-Dicarbonyl Compounds and GSH

**Figure 4:**
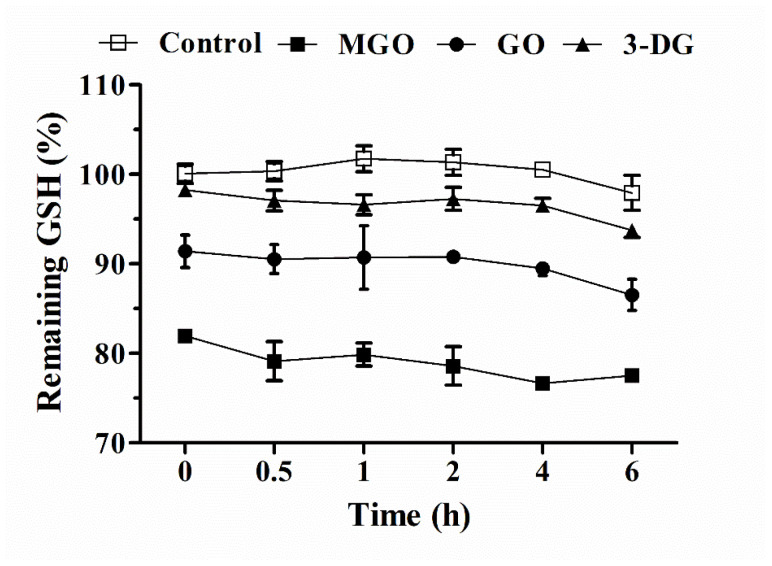
The change in GSH content after incubation of GSH (0.5 mM) with MGO (0.5 mM), GO (0.5 mM) or 3-DG (0.5 mM) in 25 mM potassium phosphate (pH = 7.4, 37 °C) for 0, 0.5, 1, 2, 4, and 6 h. Data are presented as mean ± SEM of three independent replicates.

We further evaluated and compared the kinetics of the scavenging effects of GSH on the three α-dicarbonyl compounds at a molar ratio of 1:1 in 25 mM potassium phosphate (pH = 7.4) at 37 °C. These reactions were monitored for 6 h during which the formation of GSSG from GSH was limited as confirmed by LC-TQ-MS (data not shown). The results shown in Figure 4 suggest that GSH reacted instantaneously with the three α-dicarbonyl compounds, and the scavenging of the α-dicarbonyls by GSH did not continue during the subsequent 6 h of incubation, during which only a small further decrease in GSH was observed likely due to some autoxidation of GSH. At equimolar concentrations of MGO and GSH resulted in the highest level of GSH scavenging (ca. 18.1% reduction of GSH immediately at the start), as compared with GO (ca. 8.6% reduction of GSH) and 3-DG (ca. 1.7% reduction of GSH).

### 3.3. Effects of α-Dicarbonyl Compounds on the Viability of Nrf2 CALUX Cells and Induction of Nrf2-Mediated Gene Expression

**Figure 5:**
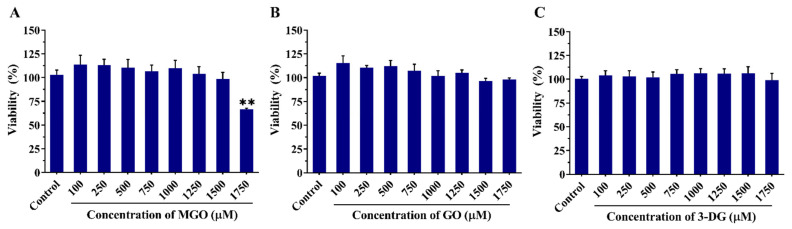
Effects of MGO (**A**), GO (**B**), and 3-DG (**C**) on the viability of Nrf2 CALUX cells as evaluated with the WST-1 assay. Data are presented as mean ± SEM of three independent replicates. ** *p* < 0.01 compared with the solvent control (0.5% nano pure water).

The WST-1 assay was used to assess the effects of MGO, GO, and 3-DG on the viability of Nrf2 CALUX cells. As shown in Figure 5, none of the individual compounds (MGO, GO and 3-DG) caused cytotoxic effects at concentrations from 100 to 1750 μM on Nrf2 CALUX cells after 24 h exposure, except for MGO that exhibited some cytotoxicity (66.6% cell viability remaining) at the maximum concentration tested (1750 μM).

**Figure 6:**
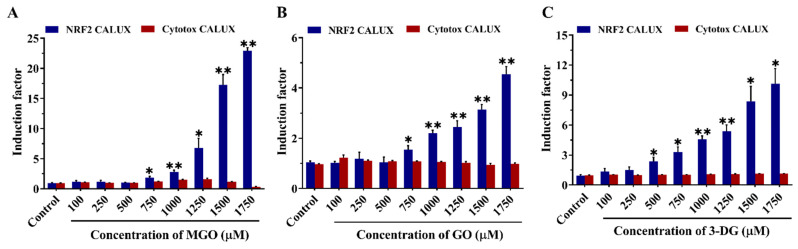
Induction of Nrf2-mediated luciferase gene expression in the Nrf2 CALUX cells (blue bars) and fold change in luciferase activity in the Cytotox CALUX assay (red bars) after 24 h exposure to MGO (**A**), GO (**B**), and 3-DG (**C**). Note the different size of the Y-axes. Data are presented as mean ± SEM of three independent replicates. * *p* < 0.05 and ** *p* < 0.01 compared with the solvent control (0.5% nano pure water).

Figure 6 shows the concentration-dependent luciferase induction by each α-dicarbonyl compound at concentrations up to 1750 μM in the Nrf2 CALUX assay and the Cytotox CALUX assay. The Cytotox CALUX results corroborate the minor effects on cell viability as also observed in the WST-1 assay for the three compounds by showing no substantial reduction (Figure 5). Furthermore, no increase in luciferase activity was observed after exposure of the Cytotox CALUX cells to the α-dicarbonyl compounds, indicating that the observed induction in the Nrf2 CALUX assay does not reflect false positive results due to the stabilization of the luciferase enzyme. The results of the Nrf2 CALUX assay presented in Figure 6 show that MGO, GO and 3-DG significantly induced Nrf2 mediated luciferase expression in a concentration-dependent manner compared to the solvent control, with the induction becoming statistically significant at concentrations ≥750 μM MGO, ≥750 μM GO, and ≥500 μM 3-DG, respectively (*p* < 0.05). 3-DG showed a luciferase induction at a lower concentration (500 µM), potentially reflecting its less efficient scavenging by GSH (Figure 4). In terms of fold induction, however, at higher concentrations (≥1250 μM) MGO appeared to be the most potent Nrf2 inducer, followed by 3-DG and lastly GO. GO showed the lowest induction capacity of Nrf2-mediated gene expression at all concentrations tested among the three compounds.

### 3.4. Effects of α-Dicarbonyl Compounds on the Production of Intracellular ROS

**Figure 7:**
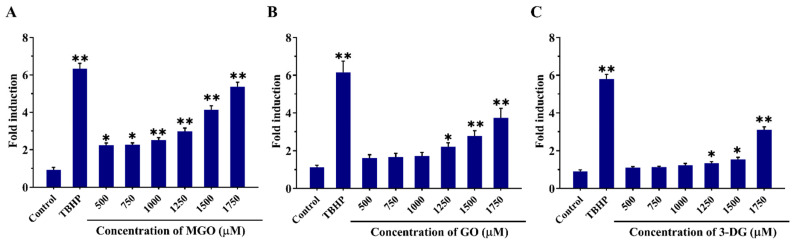
Effects of MGO (**A**), GO (**B**) and 3-DG (**C**) on the reactive oxygen species (ROS) production in Nrf2 CALUX cells. *tert*-Butyl hydroperoxide (TBHP) at 50 μM was used as the positive control. Data are presented as mean ± SEM of three independent replicates. * *p* < 0.05 and ** *p* < 0.01 compared with the solvent control.

Intracellular ROS production induced by MGO, GO and 3-DG at concentrations from 500 to 1750 μM was measured using the oxidation-sensitive probe DCFDA. As shown in Figure 7, incubation of the Nrf2 CALUX cells for 6 h with MGO, GO, and 3-DG induced a concentration-dependent increase in ROS production compared to the solvent control, with the induction becoming statistically significant at concentrations ≥500 µM MGO, ≥1250 µM GO, and ≥1250 µM 3-DG, respectively (*p* < 0.05). This effect appeared to decrease in the order MGO > GO ≈ 3-DG.

### 3.5. Effects of Altered Intracellular GSH Levels on the Viability of Nrf2 CALUX Cells and the Induction of Nrf2-Mediated Gene Expression by α-Dicarbonyl Compounds

**Figure 8:**
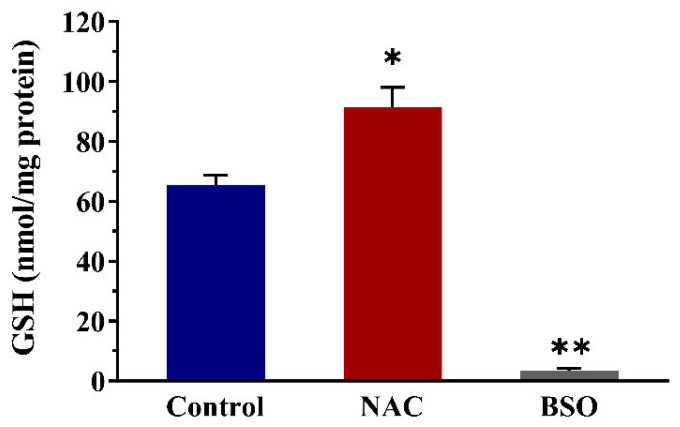
Changes in intracellular GSH levels after exposure of Nrf2 CALUX cells to NAC or BSO only. Data are shown as mean ± SEM of three independent replicates. * *p* < 0.05 and ** *p* < 0.01 compared with the solvent control. NAC: N-acetyl-L-cysteine, BSO: L-buthionine-sulfoximine.

Intracellular GSH levels were measured by LC-TQ-MS in control and NAC or BSO treated cells to validate the effectiveness in the modulation of GSH levels in the Nrf2 CALUX cells (Figure 8). After treatment with 10 mM NAC for 4 h, intracellular GSH levels were increased 1.4-fold compared to the solvent control. In contrast, intracellular GSH levels in cells treated with 100 μM BSO for 48 h were 18.4-fold lower than what was detected in untreated control cells.

**Figure 9:**
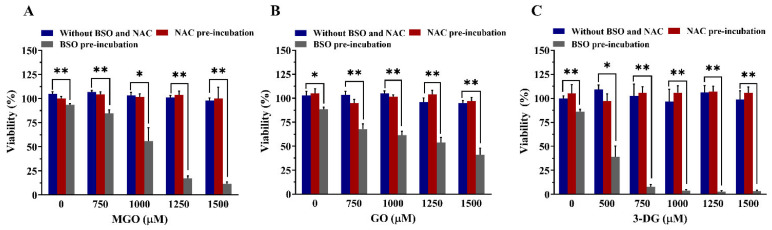
Effects of altered intracellular GSH levels on the cell viability upon exposure of Nrf2 CALUX cells to MGO (**A**), GO (**B**), and 3-DG (**C**) for 24 h. Data are presented as mean ± SEM of at least three independent replicates. * *p* < 0.05 and ** *p* < 0.01.

Figure 9 shows the effect of pre-incubation of Nrf2 CALUX cells with NAC or BSO on the cytotoxicity of the three α-dicarbonyl compounds. The selected concentrations in this assay for MGO, GO and 3-DG were not toxic to the cells with unmodified GSH levels. Upon pre-treatment of the cells with 10 mM NAC, MGO, GO, and 3-DG also showed no significant toxic effect on the cells. Treatment with BSO alone showed some cytotoxicity (89.5% cell viability remaining), while co-exposure of the cells to 100 μM BSO and the α-dicarbonyl compounds at increasing concentrations that were not toxic by themselves (Figure 5) resulted in a considerable dose-dependent additional decrease in the cell viability. Upon reduction of cellular GSH levels by BSO, MGO, GO, and 3DG showed significantly increased toxicity at concentrations that were shown non cytotoxic towards cells with unmodified GSH levels. This effect appeared to decrease in the order 3DG > MGO > GO.

**Figure 10:**
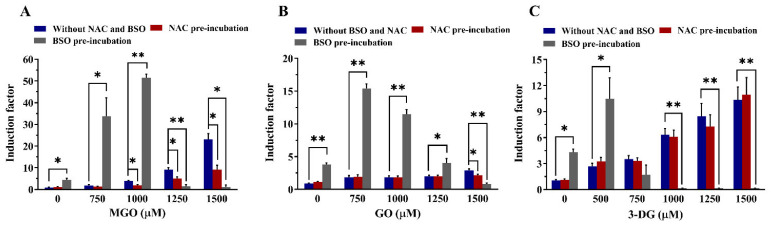
Effects of altered intracellular GSH levels on the induction of Nrf2-mediated gene expression upon 24 h exposure to MGO (**A**), GO (**B**), and 3-DG (**C**). Note the different size of the Y-axes. Data are shown as mean ± SEM of at least three independent replicates. * *p* < 0.05 and ** *p* < 0.01.

The effects of changes in intracellular GSH levels on the α-dicarbonyl-mediated induction of Nrf2-mediated gene expression are shown in Figure 10. Although treatment with NAC alone did not show any effect on basal luciferase induction, it significantly attenuated Nrf2-mediated induction of gene expression by MGO (at concentrations ≥ 1000 µM) and GO (at 1500 µM) but not 3-DG. Upon pre-treatment of the Nrf2 CALUX cells with NAC, the induction factor for the Nrf2-mediated induction of gene expression of MGO at 1000, 1250, 1500 μM decreased from 3.8- to 1.9-fold (50% decrease), 9.1- to 5.0-fold (45% decrease), and 23.1- to 9.1-fold (61% decrease), respectively, while the induction factor of GO at 1500 μM decreased from 2.9- to 2.1-fold (28% decrease). 

The addition of BSO leads to the opposite effect causing a substantial increase in luciferase induction by MGO (750 and 1000 μM), GO (750 and 1000 μM) and 3-DG (500 μM). The induction factor of MGO at 750 and 1000 μM increased from 1.9- to 33.7-fold and 3.8- to 51.5-fold, respectively. The luciferase induction of GO at 750 and 1000 μM increased from 1.9- to 15.4-fold and 1.8- to 11.5-fold, respectively. For 3-DG, an increase in induction from 2.7- to 8.9-fold at 500 μM was observed. In addition, a decrease in luciferase induction was observed at higher concentrations of the three compounds in the presence of BSO, due to the increased cytotoxic effects caused by each α-dicarbonyl compound in the presence of BSO (Figure 9). BSO itself showed an induction factor of 4.0-fold compared to the untreated control. However, the luciferase induction response by the three α-dicarbonyl compounds with the addition of BSO is considerably higher than the sum of the responses induced by each α-dicarbonyl compound and BSO separately. This indicates that reduction of cellular GSH levels by BSO resulted in a more than additive enhancement of Nrf2-mediated gene expression by the α-dicarbonyl compounds.

## 4. Discussion

**Figure 11:**
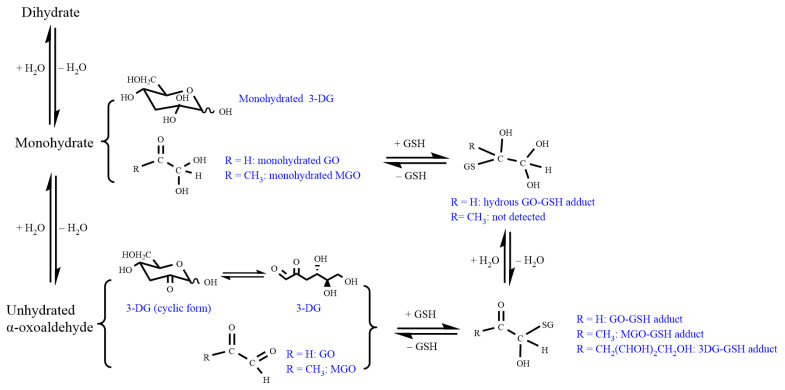
Schematic presentation of the formation of MGO-GSH, hydrous GO-GSH, GO-GSH, and 3-DG-GSH adducts.

The scavenging of GO and MGO by GSH in cells is a well-known phenomenon that occurs non-catalytically or is catalyzed by GSTs, leading to the subsequent further detoxification of GO and MGO by Glo1 and Glo2 in the glyoxalase system [7,17,34]. Our results demonstrate that 3-DG can form adducts with GSH as well. In addition, we found that, unlike what was observed for MGO, for GO also a hydrated GSH adduct was detected. The results also indicate that the three compounds react instantaneously with GSH and that the reactions reach equilibrium rapidly. In the present study, the amount of GSH scavenged by an equimolar concentration of the three α-dicarbonyl compounds decreased in the order MGO > GO > 3DG. This difference may be related to the occurrence of their various different chemical forms in aqueous solution (Figure 11) [35]. 3-DG tends to exist in a cyclic form in aqueous solutions resulting in relatively lower levels of the form that tends to react with GSH [36]. Similarly, MGO is present in aqueous solutions in a monohydrate (71%), dihydrate (28%) and unhydrated form (1%), while GO exists mainly as dihydrate followed by dimers (1–2%), monohydrate (0.5%), and an unhydrated form (0.005%) [37,38]. This difference in hydration between GO and MGO can be related to the fact that GO is a dialdehyde and therefore probably exists in doubly hydrated form, in which upon GSH adduct formation one hydrated moiety remains, while the keto group of MGO is less susceptible to hydration explaining why no hydrated adduct is formed from MGO and GSH. In addition, the fact that less unhydrated GO exists in buffer solutions and also that the monohydrate of GO tends to polymerize more rapidly than that of MGO may result in a lower level of GSH adduct formation with GO compared to MGO [38].

Apart from the primary GSH-dependent detoxification pathway through direct scavenging with or without GSTs mediation followed by the further metabolism via Glo1 and Glo2 in the glyoxalase system for MGO and GO, aldehyde dehydrogenases (ADHs) and aldo-keto reductases (AKRs) also contribute to the detoxification of MGO and GO to a lesser degree [7]. 3-DG is reported to be mainly metabolized by AKRs, with minor metabolism by ADH [18]. Despite that 3-DG did form an adduct with GSH in this study, a previous study reported that Glo1 overexpression did not result in decreased 3-DG levels and thus 3-DG was considered as not sensitive to Glo1 [39]. The expression of Glo1, AKRs, ADHs as well as the synthesis of GSH are known to be controlled by the transcription factor Nrf2 through regulatory EpRE-mediated gene expression, with the activation of Nrf2-mediated gene expression enhancing the expression of enzymes for metabolism of α-dicarbonyl compounds leading to the prevention of so-called dicarbonyl stress within cells [40]. MGO, as a highly reactive electrophilic compound is known to trigger the Nrf2-mediated adaptive cellular response [41], while studies on the induction of the Nrf2 pathway by GO and 3-DG are still absent. In the present study we compared the ability of the three α-dicarbonyl compounds to induce Nrf2-mediated gene expression using the Nrf2 CALUX reporter gene assay, which provides a powerful tool to measure Nrf2-mediated gene expression. Our results show that all three compounds are able to induce the Nrf2-mediated pathway in a dose-dependent manner, suggesting the potential role of α-dicarbonyl compounds in cellular signaling function despite the fact that they are also known as contributing factors to many diseases. It was reported that 3-DG is less reactive than MGO and GO, with for example 200-fold lower reactivity with arginine residues than MGO and GO [42], which is in line with our results that show that 3-DG has the lowest reactivity towards GSH. However, 3-DG appeared able to activate the Nrf2-mediated transcriptional program already at somewhat lower concentrations compared to MGO and GO. In terms of fold induction, at higher concentrations (≥1250 µM) the induction factors for the three dicarbonyls decreased in the order MGO > 3DG > GO. One possible explanation is that 3-DG was less efficiently scavenged by GSH, resulting in higher cellular concentrations, and thus induction of Nrf2-mediated gene expression at a lower concentration, while the potential of MGO to activate Nrf2 pathway is higher. Besides, due to the high abundance and kinetic constants of Glo1 for the metabolism of MGO and GO in comparison to the essential detoxifying enzymes AKRs for 3-DG [43], a slower metabolism of 3-DG in cells can be expected which may also result in the higher induction capacity of 3-DG as compared to GO. In addition, the underlying mode of action by which the three α-dicarbonyl compounds mediate Nrf2 induction may be related to either (i) ROS production and/or (ii) their electrophilicity and reaction with amino acid moieties of Keap1 resulting in the release of Nrf2. With respect to ROS production, these three compounds were shown in the present study to be able to induce ROS production, with the fold induction decreasing in the order MGO > GO ≈ 3-DG. In addition, α-dicarbonyl compounds may react reversibly with cysteine residues of Keap1, and/or may also form irreversible adducts with other nucleophilic amino acid residues such as arginine and lysine, leading to the Nrf2 release. A previous study discovered that MGO was able to modify Keap1 to form a crosslinking AGE between a cysteine and arginine residue of Keap1, resulting in the dimerization of Keap1 followed by the release of Nrf2 and activation of the Nrf2 transcriptional program [44]. The irreversible adduct formed by MGO may cause prolonged activation of Nrf2 in comparison to reversible binding to its cysteine moieties. Therefore, the reactivity of dicarbonyls towards GSH may not be the only indicative parameter of their Nrf2 induction capacity.

We further studied the role of intracellular GSH levels in the Nrf2-mediated gene expression. The intracellular GSH levels were modulated by the addition of NAC and BSO, which resulted in 1.4-fold increase and 18.4-fold decrease in cellular GSH levels, respectively. This difference in the degree of intracellular GSH level modulation may possibly explain the more pronounced influence on the Nrf2-mediated luciferase induction by the α-dicarbonyl compounds upon the addition of BSO than upon pre-treatment with NAC in our cell model. Pre-treatment of the cells with NAC to increase intracellular GSH levels resulted in a decrease in the Nrf2-mediated gene expression by MGO and GO, but not by 3-DG. This difference is ascribed to the lower level of scavenging of 3-DG with GSH, for which apparently available levels of GSH are adequate and a further increase in cellular GSH levels does not provide extra protection. However, the addition of BSO to decrease the intracellular GSH levels resulted in enhanced cytotoxicity as well as increased induction of Nrf2-mediated gene transcription by MGO, GO and also by 3-DG. The effects of GSH depletion on the Nrf2 induction by the three compounds appeared to decrease in order of MGO > GO > 3-DG. These results indicate the difference in the role of GSH in the protection against cytotoxicity and in the activation of Nrf2-mediated gene expression by MGO, GO and 3-DG. For MGO and GO, the depletion of cellular GSH can be expected to result in the inhibition of the formation of the hemithioacetal via reaction of MGO and GO with GSH, leading to less efficient detoxification of MGO and GO in the cells, thereby increasing their cytotoxicity and Nrf2 inducing activity. In the meanwhile, the depletion of GSH may also increase the oxidative stress in the cells which may contribute to the increased cytotoxic effects and Nrf2 inducing activity. For 3-DG, the enhanced Nrf2 induction activity may be mainly due to the increased oxidative stress in cells caused by 3-DG and the depletion of GSH. A quite unexpected finding was the highest cytotoxic effects by 3-DG in the presence of BSO. Besides the increased oxidative stress which may contribute to the cytotoxic effects to some extent, another possible explanation for the results is that the depletion of GSH may lead to the decrease in the cellular NADPH levels in the regeneration of GSH from GSSG in oxidative stress, which would further cause the decrease of the activity of AKRs leading to the inhibition of the detoxification of 3-DG in the cells [18,45]. The exact mode of action underlying this discrepancy for 3-DG, however, remains to be elucidated.

α-Dicarbonyl compounds have traditionally been considered as toxic metabolites leading to pathological disorders and many diseases [7,40]. However, some recent studies proposed that a hormetic effect can be induced by lower levels of reactive carbonyl species resulting in biological beneficial effects [46]. In a healthy human body, a lower level of dicarbonyls may induce adaptive responses such as induction of Nrf2 mediated gene expression leading to the prevention or repair of the adverse effects of the dicarbonyls. However, overwhelming of the protective mechanisms by elevated levels of dicarbonyls and accompanying decreased GSH levels may play a role in some chronic diseases for example neurodegenerative diseases and diabetes [47,48]. The augmented and persistent activation of the Nrf2 pathway by dicarbonyl stress can be considered to counteract the related potential adverse effects at least to some extent.

By using U2OS reporter cell lines, our study revealed the potential of the selected α-dicarbonyl AGE precursors to activate Nrf2 mediated gene expression and the role of intracellular GSH in counteracting this effect. The results also revealed subtle differences between the three α-dicarbonyls with MGO being the most potent in terms of scavenging by GSH, ROS production, induction of Nrf2-mediated gene expression, and the effects of GSH depletion on this potential. It is of interest to note that other studies report the toxicity of MGO to other cell lines [22,49]. Comparison of the toxicity data from these studies to the results of the present study reveals that U2OS cells appear less sensitive to the adverse effects of MGO. Nevertheless, these cells were used to create the Nrf2 reporter cell model because generally U2OS cells are considered suitable for this purpose given their lack of high levels of endogenous receptors, reducing chances on receptor cross talk [23,50]. For all of these in vitro toxicity data reported in the present study it holds that the concentrations of the dicarbonyls at which they cause toxicity are beyond expected physiological concentrations. Nevertheless, the results of the present study reveal information relevant for the mode of action underlying cellular effects of these dicarbonyls and show that endogenous GSH levels play a role in the protection against dicarbonyl stress. Future studies in cell lines representing tissues known to be sensitive towards glycation products and/or dicarbonyl stress, for example kidney or neuronal cells [51,52], may use different or additional read outs, such as PCR or proteomics, to quantify the induction of Nrf2-mediated gene and protein expression. The results of the present study reveal that for such studies MGO is the preferred model compound.

In conclusion, this study elucidated subtle differences in the induction of Nrf2-mediated gene expression by the three typical α-dicarbonyl compounds and their scavenging by GSH, and pointed at a dual biological effect of the α-dicarbonyl compounds, being reactive toxic electrophiles and -as a consequence- able to induce Nrf2-mediated protective gene expression, with MGO being the most reactive.

## Data Availability

The data presented in this study are available on request from the corresponding author.
